# Transient Resetting: A Novel Mechanism for Synchrony and Its Biological Examples

**DOI:** 10.1371/journal.pcbi.0020103

**Published:** 2006-08-04

**Authors:** Chunguang Li, Luonan Chen, Kazuyuki Aihara

**Affiliations:** 1 ERATO Aihara Complexity Modelling Project, Komaba Open Laboratory, University of Tokyo, Tokyo, Japan; 2 Institute of Industrial Science, University of Tokyo, Tokyo, Japan; 3 Centre for Nonlinear and Complex Systems, University of Electronic Science and Technology of China, Chengdu, People's Republic of China; 4 Department of Electrical Engineering and Electronics, Osaka Sanyo University, Osaka, Japan; University College London, United Kingdom

## Abstract

The study of synchronization in biological systems is essential for the understanding of the rhythmic phenomena of living organisms at both molecular and cellular levels. In this paper, by using simple dynamical systems theory, we present a novel mechanism, named *transient resetting,* for the synchronization of uncoupled biological oscillators with stimuli. This mechanism not only can unify and extend many existing results on (deterministic and stochastic) stimulus-induced synchrony, but also may actually play an important role in biological rhythms. We argue that transient resetting is a possible mechanism for the synchronization in many biological organisms, which might also be further used in the medical therapy of rhythmic disorders. Examples of the synchronization of neural and circadian oscillators as well as a chaotic neuron model are presented to verify our hypothesis.

## Synopsis

Synchronization of dynamical systems is a process whereby two or more systems adjust a given property of their motions to a common behavior due to coupling or forcing. Synchronization has attracted much attention from physicists, biologists, applied mathematicians, and engineers for many years and is a ubiquitous phenomenon. In this paper, Li et al. present a very simple, but general mechanism, called *transient resetting,* that explains stimulus-induced synchronization in dynamic systems. The mechanism pertains not only to periodic oscillators but also to chaotic ones, and not only to continuous time systems but also to discrete time systems. Biological systems are dynamic, and their synchronization is essential, for example, in the genesis of rhythmic phenomena and information processing. In this paper, the authors study several possible instances of their novel mechanism in a biological context. They also suggest that transient resetting might be used therapeutically in rhythmic disorders. The beneficial role of noise in biological systems has been studied extensively in recent years. Li and colleagues' mechanism provides an explanation for this role in the synchronization in biological systems, even when the stimulus or input to the system is not random or noisy.

## Introduction

Life is rhythmic. Winfree made the shocking discovery that a stimulus of appropriate timing and duration can reset (stop) the biological rhythm by driving the clock to a “phase singularity,” at which all the phases of the cycle converge and the rhythm's amplitude vanishes. He theoretically predicted this in the late 1960s, and then confirmed it experimentally for the circadian rhythm of hatching in populations of fruitflies. Subsequent studies have shown that mild perturbations can also quench other kinds of biological oscillations; for example, breathing rhythms and neural pacemaker oscillations [1,2]. Such findings may ultimately have medical relevance for disorders involving the loss of a biological rhythm, such as sudden infant death syndrome or certain types of cardiac arrhythmias [3]. In [4], Tass studied phase resetting by using methods and strategies from synergetics. In [5], Leloup and Goldbeter presented an explanation for this kind of long-term suppression of circadian rhythms by the coexistence of a stable periodic oscillation and a stable steady state in the bifurcation diagram.

On the other hand, synchronization is essential for biological rhythms and information processing in biological organisms. So far, many researchers have studied the synchronization in biological systems experimentally, numerically, and theoretically. In this paper, by using simple dynamical systems theory, we show that transient resetting, which concept will be clarified later, can play a constructive role for biological synchrony. We argue that transient resetting is a possible mechanism for synchrony generally used in many biological organisms, which might also be further used in the medical therapy of rhythmic disorders. Winfree's results showed the destructive aspect of the resetting, while we show its constructive aspect. Although we concentrate mainly on biological systems, the novel mechanism presented in this paper is applicable to general oscillators, so in the following we first present it as a general mechanism for synchrony and then discuss its possible applications to biological rhythms.

## Results

### Basic Mechanism

Let's assume that an oscillator has a bifurcation diagram like that shown in Figure 1 with a normal (supercritical) Hopf bifurcation [6], in which the bifurcation parameter *λ* is the above-mentioned “stimulus,” and the curves with *λ* < *λ*_0_ show the maximum and minimum values of the oscillator state. When the stimulus is a constant less than the critical value (bifurcation point) *λ*_0_, the system has a stable rhythm (periodic oscillation); when the stimulus *λ* > *λ*_0_, the system has a stable equilibrium state, which is shown by the curve *λ* > *λ*_0_. Undoubtedly, many oscillators have this kind of bifurcation structure. Assume that a population of (identical) oscillators operates in stable periodic states with *λ = a*. To clarify the essential role of transient resetting, we don't consider coupling among oscillators in this paper. For oscillators without coupling, the situation for studying the synchronization of two oscillators is the same as that for a population of oscillators, so in the following we always consider two oscillators. Let us also assume that there are some common fluctuations, e.g., periodic fluctuations or random noise, that can perturb the parameter *λ* from *a* to the right side of *λ*_0_, say to *λ = b* in Figure 1, from time to time. When the duration of the oscillators staying to the right of *λ*_0_ is long enough, the two oscillators will all converge to the steady state, which is a rhythm-vanishing phenomenon. Next, let us examine what will happen when the parameter *λ* visits the value *λ = b* for a short duration. If the states of the two oscillators are at points *A* and *B,* respectively, the two oscillators will have the tendency to converge to their common steady state when the parameter *λ* visits the value *λ = b*, which means that the states of the two oscillators will have the tendency to become closer. If the states of the two oscillators are at the points *A* and *C,* respectively, the two oscillators will also have the tendency to converge to their common steady state when the parameter *λ* visits the value *λ = b*. From conventional linear stability analysis, we know that the velocity for converging to the steady state at point *C* is higher than that at point *A,* so the two oscillators will also have the tendency to become closer. Thus, by a short visit to the right of *λ*_0_, the states of the two oscillators always have the tendency to become closer, which is helpful for the genesis of synchronization between the oscillators. Here, the states of the oscillators are not really reset to the steady state, but they have the tendency (for a short time) to be reset to the steady state, which is the reason we call it *transient resetting*.

**Figure 1:**
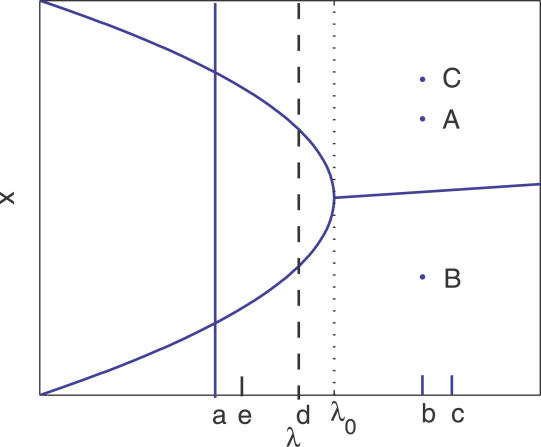
A Schematic Bifurcation Diagram with a Normal (Supercritical) Hopf Bifurcation for Illustrating the Transient Resetting Mechanism

From the above analysis, it is easy to see that the bifurcation is not necessarily required to be a supercritical Hopf bifurcation. We only need the oscillator to operate mostly in an oscillatory state, and it can from time to time visit a steady state in the parameter space. Even if there are other bifurcations between these two states (*λ = a* and *λ = b*), say a subcritical Hopf bifurcation with coexistence of a stable limit cycle and a stable equilibrium state between the dashed and the dotted vertical lines in Figure 1, the above argument can still hold. Moreover, since oscillators are usually nonidentical in real systems, there are some mismatches between the oscillators. If the mismatches are not so large, however, the oscillators can also be synchronized (although not perfectly) by transient resetting. For example, we assume that the mismatch can be reflected in the parameter *λ,* and we assume that the two oscillators operate with parameters *λ = a* and *λ = e,* respectively, and, by a common perturbation, the parameters of the two oscillators visit *λ = b* and *λ = c,* respectively, in Figure 1. If the mismatch between the systems is not so large, the distance between the steady states with *λ = b* and *λ = c* is most likely small, too. The two oscillators have the tendency to be contracted to the two steady states, respectively, which means that roughly they are becoming closer since the two steady states are close. In transient resetting, we do not care what the stimulus is. It can be of any kind, say periodic, random, impulsive, or even chaotic stimuli; thus, transient resetting can unify many existing results on stimulus-induced synchrony.

Next, we present several examples of biological rhythms to show the effectiveness of transient resetting and its biological plausibility as a mechanism for biological synchrony.

### Reliability of Neural Spike Timing

A remarkable reliability of spike timing of neocortical neurons was experimentally observed in [7]. In the experiments, rat neocortical neurons are stimulated by input currents. When the input is a constant current, a neuron generates different spike trains in repeated experiments with the same input. It is evident that the constant input when viewed as a bifurcation parameter has moved the neuron dynamics from a steady state into a repetitive spiking region. It is shown that when Gaussian white noise is added to the constant current, the neuron generates almost the same spike trains in repeated experiments. From the viewpoint of synchronization, the repeated firing patterns imply that a common synaptic current can induce almost complete synchronization in a population of uncoupled identical neurons with different initial conditions. This kind of synchronization may have great significance in information transmission and processing in the brain (see, e.g., [8–12]).

We simulate the above-mentioned behavior by using the well-known Hodgkin-Huxley (HH) neuron model, which is described by the following set of equations [13]:

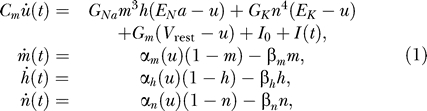
where *u*(*t*) represents the membrane potential of the neural oscillator, *m*(*t*) and *h*(*t*) the activation and the inactivation of its sodium channel, *n*(*t*) the activation of the potassium channel, *I*_0_ the constant input current, and *I*(*t*) the time-varying forcing. *α_x_* and *β_x_* (*x* = *m*, *h*, *n*) are rate functions that are given by the following equations:


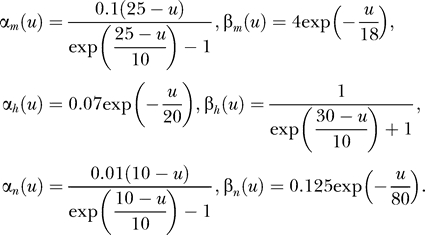


The parameters are set as the standard values [13], i.e., *G_Na_* = 120 mS/cm^2^, *E_Na_* = 115 mV, *G_K_* = 36 mS/cm^2^, *G_m_* = 0.3 mS/cm^2^, *V*_rest_ = 10.6 mV, and *C_m_* = 1 *μ*F/cm^2^. We always set the input *I*_0_ + *I*(*t*) to zero for *t* < 0. When *I*(*t*) = 0, if the value *I*_0_ is larger than a critical value 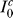 ≈ 6 *μ*A/cm^2^, the neuron has regular spiking; otherwise, the neuron is in the steady resting state. Thus, the bifurcation direction of the HH neuron is opposite to that shown in Figure 1 and the type of Hopf bifurcation is subcritical rather than supercritical. We fix the input constant *I*_0_ = 10, such that when *I*(*t*) = 0 (*t* > 0), this model exhibits a stable periodic oscillation.

To simulate the experimental results in [7], we let *I*(*t*) = *Dξ*(*t*) in the HH model, where *D* is a positive constant, and *ξ*(*t*) is a normal Gaussian white noise process with mean zero and standard deviation one. We numerically calculate the stochastic neuron model by using the Euler-Maruyama scheme [14] with time step *h* = 0.01. When *D* = 2, the two HH neural oscillators can achieve complete synchronization as shown in Figure 2A. One may ask whether it is really the “noisy” property of *I*(*t*) that synchronizes the oscillators. We show here that this behavior can be interpreted by the transient resetting mechanism. In fact, when *I*(*t*) is a random noise, it will make the total input *I*_0_ + *I*(*t*) visit the parameter region that is smaller than the critical value 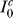 ≈ 6 from time to time, so that the transient resetting mechanism can take effect. In the experiments, the total input is indeed below the critical value in some time durations, which implies that our argument is biologically plausible. The experimental (and simulation) results show that the larger the noise intensity (to some extent), the higher the reliability and precision of the spike timing, which can also be interpreted by the transient resetting mechanism. In fact, when the noise intensity is larger, the total input will spend more time below 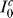 , such that the two trajectories will have more opportunities to be contracted together. Due to the random property of the input, when the input value is above 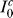 , the effects of the noise for converging and diverging the two trajectories are roughly balanced; thus, the longer the duration of the input value being below 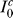 , the closer the two trajectories and the faster the convergence should be. To further verify the above argument that it is not the “noisy” property of the input that synchronizes the oscillators, we perform another simulation, in which the fluctuation is a square wave with amplitude of 4.5 and period of 20 ms; that is, the total input switches between 10 (above 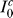 ) and 5.5 (a little below 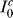 ) every 10 ms. In Figure 2B, we show the simulation result, in which the square wave is added at *t* = 80. We see that the two neural oscillators are synchronized rapidly, though the input is a regular square wave.

**Figure 2:**
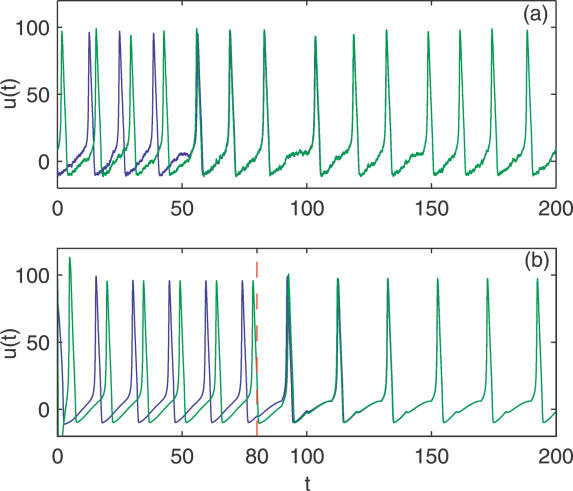
Synchronization of Two HH Neural Oscillators (A) Driven by common Gaussian white noise; and (B) driven by a common square wave.

Next, we numerically study the relationship between the time rate that the stimulus of the HH neuron model spends in the steady-state region (defined as the *duty*) and the time required to achieve synchronization. For the convenience of measuring the duty, we use a square wave as the stimulus in the simulations. In the following simulation, the period of the square wave is 20 ms, the time window when the stimulus is in the steady-state parameter region is randomly chosen in each cycle, and all the other parameters are the same as those in the above simulation. In Figure 3, we plot the relationship between the duty and the time required to achieve synchronization as the duty increases from 0.1 to 0.4 with step 0.05, in which the data are obtained by averaging the results in 20 independent runs. Figure 3 shows that the time required to achieve synchronization decreases as the duty increases. This quantitative result again confirms our above argument and provides numerical evidence that the proposed mechanism can account for the observed synchrony.

**Figure 3:**
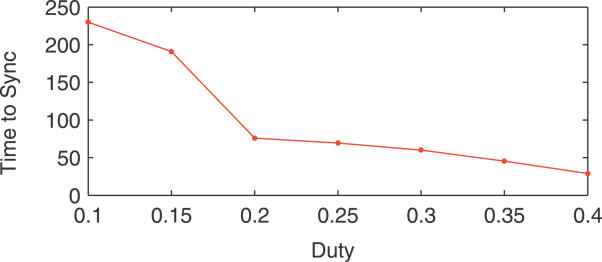
The Relationship between the Duty and the Time Required to Achieve Synchronization in the HH Neuron Model

Biological oscillators are usually nonidentical in real systems, and there are some mismatches between the oscillators. As we mentioned in the Basic Mechanism section, if the mismatches are not so large, the oscillators can also be synchronized (although not perfectly) by transient resetting. We next numerically study the relationship between the parameter mismatch and the synchronization error, that is, the robustness of the mechanism, in the HH systems. In this simulation, we consider the mismatch on *I*_0_ in Equation 1, namely in one of the two neural oscillators, the constant input is *I*_0_ − Δ*I*, and in the other one, it is *I*_0_. As we know, the input value *I* has explicit effect on the spiking frequency of the HH neuron model. We also use a square wave in this simulation, and the period and the duty are 10 and 0.3, respectively. Again, the time window when the stimulus is in the steady-state parameter region is randomly chosen in each cycle. The synchronization error is defined as *q* = <| *X*_1_ (*t*) − *X*_2_ (*t*) |>, where *X*_1_, *X*_2_ are the state vectors of the two neural oscillators, that is, *X_i_*(*t*) = [*u_i_*(*t*),*m_i_*(*t*),*h_i_*(*t*),*n_i_*(*t*)]*^T^*, *i* = 1, 2, and < · > is the average over time after discarding the initial phase of the simulation for 100 ms. In Figure 4A, we plot the relationship between Δ*I* and *q,* in which each value of *q* is also obtained by averaging the results in 20 independent runs and the error bars denote the standard deviations. Figure 4A shows that the synchronization error *q* increases with the increasing of Δ*I,* and when Δ*I* is not so large, the systems can also achieve synchronization with a small synchronization error *q*. In Figure 4B, we plot a typical simulation result with Δ*I* = 0.5, and the *q* value between 100 ms and 200 ms in this simulation is *q* = 3.8667. Figure 4B demonstrates that the transient resetting mechanism can indeed make the two nonidentical neural oscillators synchronous, though the synchrony is not perfect. When the mismatch becomes large, the systems may intermittently lose synchrony, but shortly after that the systems can be attracted back to synchrony again by the mechanism. When the mismatch becomes much larger, the systems cannot maintain the synchronous state, though the mechanism still has the tendency to draw the systems together. In biological systems, the oscillators, though not perfectly identical, can usually be similar and the mismatch may not be so large. Thus, the presented mechanism is robust in such cases. Moreover, the synchronization in biological systems may not be required to be perfect for the emergence of functions.

**Figure 4:**
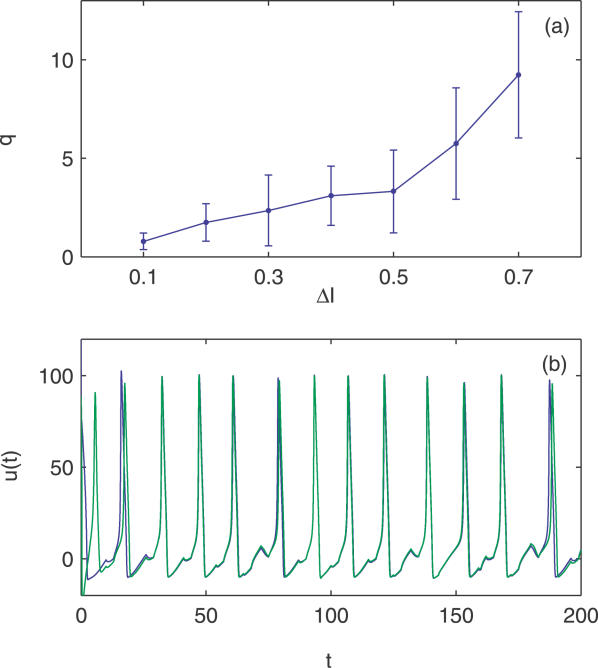
Robustness of the Mechanism with Parameter Mismatch on *I*_0_ in the HH Neuron Model (A) The relationship between the parameter mismatch Δ*I* and the synchronization error *q*. (B) A typical numerical result of the systems with Δ*I* **=** 0.5.

It should be noted that when the mismatches exist in other parameters except the input, we can also obtain similar results. For example, in another simulation, we consider a mismatch on the parameter *G_m_*, that is, the parameter is *G_m_* + Δ*G_m_* in one oscillator, and it is *G_m_* in the other oscillator. The simulation methods are the same as above. In Figure 5A, we show the relationship between Δ*G_m_* and *q* as Δ*G_m_* increases from 0.02 to 0.1 with step 0.02. In Figure 5B, we plot a typical numerical result with Δ*G_m_* = 0.06, and the *q* value between 100 ms and 200 ms in this simulation is *q* = 2.1492. From this figure, we can get the same conclusion as that in the above example.

**Figure 5:**
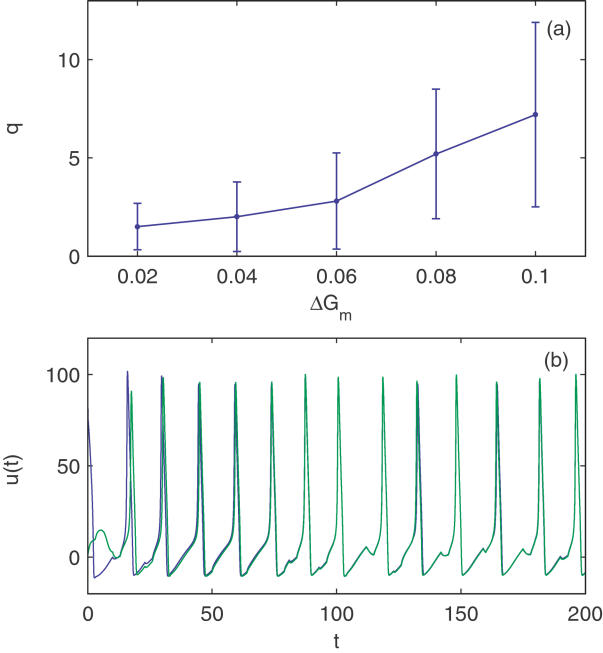
Robustness of the Mechanism with Parameter Mismatch on *G_m_* in the HH Neuron Model. (A) The relationship between the parameter mismatch Δ*G_m_* and the synchronization error *q*. (B) A typical numerical result of the systems with Δ*G_m_* **=** 0.06.

It should also be noted that this transient resetting is effective for both class I and class II neurons [13]. Further, the Hopf bifurcation of class II neurons can be either subcritical as in the HH model, or supercritical associated with the canard phenomenon [15].

### Circadian Oscillators

In circadian systems, the light–dark cycle is the dominant environmental synchronizer used to entrain the oscillators to the geophysical 24-h day. In the following, we show that the light–dark cycle as a synchronizer can also be interpreted by the transient resetting mechanism. By our argument on transient resetting, if the circadian clock has a bifurcation diagram similar to Figure 1 with a light-affected parameter as the bifurcation parameter, and if the oscillators operate in the oscillatory parameter region (say with *λ* = *a* in Figure 1) in the dark duration, and in the steady-state parameter region (say with *λ* = *b* in Figure 1) in the light duration, the oscillators may be automatically synchronized. For example, in the Leloup-Goldbeter model of *Drosophila* [16], the light–dark cycle affects the degradation of the TIM protein. In their parameter setting (which is biologically plausible), under a continuous light condition, the model is in the steady-state parameter region. Then, according to the transient resetting mechanism, the uncoupled oscillators can be synchronized automatically, which is verified by our numerical simulations (unpublished data).

In theoretical studies of circadian clocks, the light–dark cycle is usually represented by a square wave, but, in fact, even with continuous light, the light intensity includes fluctuations (light noise). Next, we study the effect of light noise on the synchronization of circadian oscillators. We consider the Goldbeter circadian clock model of *Drosophila* [17] as an example, which is described as follows:

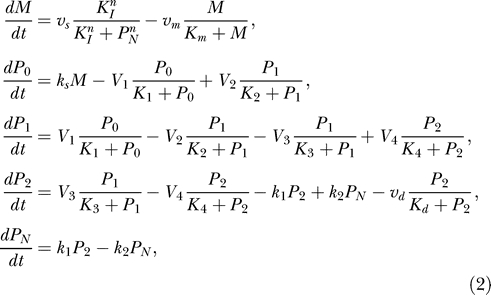
where the parameter values are *v_s_* = 0.76 *μ*Mh^−1^, *v_m_* = 0.75 *μ*Mh^−1^, *K_m_* = 0.5 *μ*M, *k_s_* = 0.38 h^−1^, *v_d_* = 1 *μ*Mh^−1^, *k*_1_ = 1.9h^−1^, *k*_2_ = 1.3h^−1^, *K_I_* = 1 *μ*M, *K_d_* = 0.2 *μ*M, *n* = 4, *K*_1_ = *K*_2_ = *K*_3_ = *K*_4_ = 2 *μ*M, *V*_1_ = 3.2 *μ*Mh^−1^, V_2_ = 1.58 *μ*Mh^−1^, *V*_3_ = 5 *μ*Mh^−1^, and V_4_ = 2.5 *μ*Mh^−1^ (see [17] for more details about this model). In this model, light enhances the degradation of the PER protein by increasing the value of *v_d_*. With these biologically plausible parameter values, the period of the circadian clock is about 24 h. With the increasing of *v_d_,* there is a Hopf bifurcation similar to that shown in Figure 1, and the critical value of *v_d_* at the bifurcation point is about 1.6. To clarify the effect of light noise, we don't consider the light–dark cycle in this simulation, that is, *v_d_* = 1 is a constant (denoting the average light intensity) if there is no light fluctuation. In our simulation, we set *v_d_* = 1 + *Dξ* (*t*). When *D* = 0.15, the simulation result is shown in Figure 6A, which shows that the two circadian oscillators achieve complete synchronization. This behavior can again be interpreted by the transient resetting mechanism. The interpretation is the same as that in the HH neural oscillator case.

**Figure 6:**
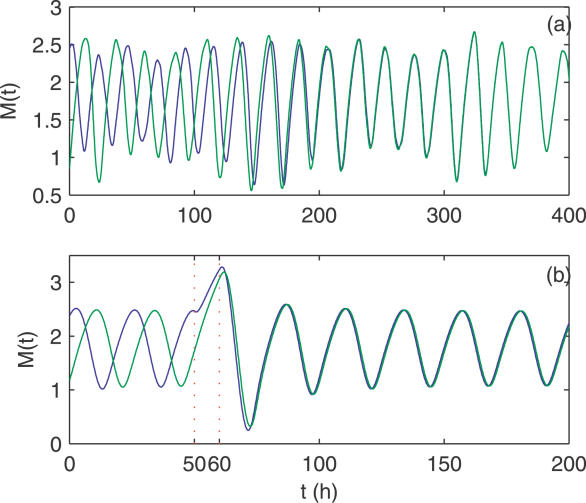
Synchronization of Two Goldbeter Circadian Oscillators (A) Induced by common light noise; and (B) induced by a short duration of bright light.

In the above examples, we show separately the effects of the light–dark cycle and the light noise. The light–dark cycle itself may not be strong enough to reach the steady-state parameter region in real biological circadian systems. For example, in Figure 1, the light–dark cycle may make the bifurcation parameter switch between *a* and *d* in the dark and the light durations, respectively. In this case, a small light noise would drive the parameter *λ* to the right hand side of *λ*_0_ from time to time (in the light duration), which can be seen as the combination or synergetic effect of the light–dark cycle and the light noise. In other words, in biological circadian systems, it is likely that the light–dark cycle and the light noise cooperate to realize transient resetting.

The time required to achieve synchrony and the robustness of the mechanism in this system can also be studied similarly as in the HH model, although we omit the detailed results here.

### Therapy

The transient resetting mechanism may have potential applications in the therapy of various rhythmic disorders. Our analysis implies that if we have some methods to control a biological rhythmic system to make it visit its steady-state parameter region transiently, it may entrain the disordered rhythmic system to a synchronous state. For example, we use a stimulation bright light for 10 h with *v_d_* = 1.7 in ∈ [50,60], and *v_d_* = 1 in other time durations in the Goldbeter circadian oscillators. The simulation result is shown in Figure 6B, which shows that the two oscillators are almost completely synchronized after the short duration of the bright light stimulation. The exposure to bright light also induces a several-hour delay shift of the circadian oscillators, which is consistent with the experimental results [18].

Excepting the above-mentioned neural and circadian systems, the mitotic control system [19] may be another biological example that uses the transient resetting mechanism to achieve synchrony.

### Chaotic Neuron Model

In the above examples, we considered periodic oscillators, but transient resetting, as a general mechanism for synchrony, can also be applied to chaotic systems. Clearly, the synchronization of chaotic systems is more difficult, because uncoupled chaotic oscillators, even with identical parameter values, will exponentially diverge due to the high sensitivity to perturbations. If the converging effect in the steady-state parameter region is larger than the diverging effect in the chaotic parameter region, however, the uncoupled chaotic systems may also be synchronized by the mechanism. Here, we consider a simple discrete-time chaotic neuron model described as follows [20]:

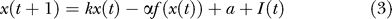
where *f*(*x*(*t*)) = 1/[1 + exp(−*x*(t)/*ɛ*)]. This neuron model is a model of the chaotic responses electrophysiologically observed in squid giant axons [21]. When decreasing *a* from a large to a small value, the system undergoes a period-doubling road to chaos. We set the parameters *k* = 0.7, *α* = 1.05, *a* = 0.93, and *ɛ* = 0.02, such that when *I*(*t*) = 0 the model exhibits chaotic dynamics. When *I* > 0.11, the neuron model is in a steady state. In this example, we let I(*t*) = 0.15*ξ*(*t*) with *ξ*(*t*) as a normal Gaussian white noise process as defined before, such that the neuron model can switch between chaotic states and steady states. The numerical result in Figure 7 indicates that the chaotic neurons can indeed be synchronized by common noise, which can also be interpreted by the transient resetting mechanism.

**Figure 7:**
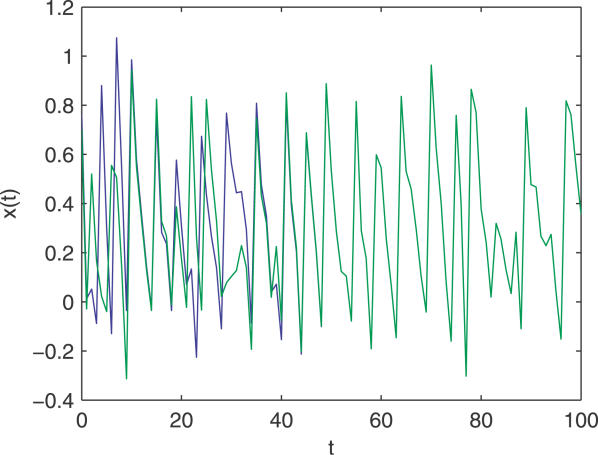
Synchronization of Two Chaotic Neurons Induced by Common Noise

This example, though simple, shows that the transient resetting mechanism pertains not only to periodic oscillators but also to chaotic systems, and not only to continuous time systems but also to discrete time systems. We can also see that, as we mentioned above, the bifurcation is not necessarily required to be a Hopf bifurcation.

## Discussion

In the literature, there exist some interesting results on noise-induced synchronization of oscillators, which can be classified into two classes: the noise does or does not depend on the states of the oscillators. In the case that the noise depends on the states of the oscillators (see, e.g., [22,23]), the noise can, in fact, be seen as a kind of information exchange or coupling with fluctuant coupling strengths, and it is well-known that coupling can induce synchronization, so it is not surprising that the noise can induce synchronization. In the case that noise does not depend on the states, many existing results can be interpreted by transient resetting. Some studies in the literature also theoretically explained the mechanisms of periodic input forcing induced synchrony. It should be noted that the fluctuations in the present mechanism of transient resetting are the parameters, not necessarily (but can be) the inputs. Synchronization induced by the fluctuations of some special parameters, for example time delay in delayed systems, can also be interpreted by the mechanism. Thus, transient resetting not only can unify and extend many existing results of various fluctuation-induced synchrony, but also is very simple. It is reasonable to believe that life systems, after a long time of evolution, use mechanisms that are as simple as possible to achieve complex functions.

It should also be noted, on the other hand, that although we have shown transient resetting as a possible general mechanism for biological synchrony, we don't exclude other possible mechanisms. Some systems driven by some *specific* inputs, which don't satisfy the conditions shown in this paper, can also be synchronized. It should not be surprising that in biological systems many mechanisms work together to jointly guarantee the robustness and precision of synchrony.

In summary, by simple dynamical systems theory, we have presented a novel mechanism for synchrony based on transient resetting, and we have shown that it could be a possible mechanism for biological synchrony, which can also potentially be used for medical therapy. In contrast to Winfree's results, we have shown the constructive aspect of (transient) resetting. In this paper, we are interested in the general qualitative mechanism, so in the simulations we didn't show many quantitative details for each specific example. In Figure 1, we showed a one-parameter bifurcation diagram. In some systems, there might be multiple parameters that are affected by the fluctuations of stimuli. In that case, we can use a similar multiparameter bifurcation diagram to understand the mechanism.

## Materials and Methods

To simulate the stochastic differential equation 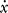 = *f*(*x*) + *g*(*x*)*ξ*(*t*), the Euler-Maruyama scheme is used in this paper. In this scheme, the numerical trajectory is generated by *x_n_*_+1_ = *x_n_* + *hf*(*x_n_*) +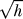 *g*(*x_n_*)*η_n_*, where *h* is the time step and *η_n_* is a discrete time Gaussian white noise with < *η_n_* > = 0 and < *η_n_ η_m_* > *δ_nm_*. For more details, see, e.g., [14].

## Abbreviation

HH: Hodgkin-Huxley
